# The Prevalence of and Risk Factors Associated with Musculoskeletal Disorders in Thai Oil Palm Harvesting Workers: A Cross-Sectional Study

**DOI:** 10.3390/ijerph18105474

**Published:** 2021-05-20

**Authors:** Petcharatana Bhuanantanondh, Bryan Buchholz, Sara Arphorn, Pornpimol Kongtip, Susan Woskie

**Affiliations:** 1 Faculty of Physical Therapy, Mahidol University, 999 Phuttamonthon 4 Road, Salaya, Nakhon Pathom 73170, Thailand; 2 Department of Biomedical Engineering, Francis College of Engineering, University of Massachusetts Lowell, One University Ave, Lowell, MA 01854, USA; Bryan_Buchholz@uml.edu; 3 Department of Occupational Health and Safety, Faculty of Public Health, Mahidol University, 420/1 Rajvithi Road, Bangkok 10400, Thailand; sara.arp@mahidol.ac.th (S.A.); pornpimol.kon@mahidol.ac.th (P.K.); 4 Department of Public Health, Zuckerberg College of Health Sciences, University of Massachusetts Lowell, 61 Wilder St., Lowell, MA 01854, USA; Susan_Woskie@uml.edu

**Keywords:** musculoskeletal disorders, oil palm workers, harvester, ergonomics

## Abstract

Musculoskeletal disorders (MSDs) are common in various occupations. However, there is still limited research about the prevalence of, and risk factors associated with, MSDs among oil palm harvesting workers in Thailand. To investigate the prevalence of MSDs and risk factors associated with MSDs in Thai oil palm harvesting workers, face-to-face interviews were conducted with Thai oil palm harvesting workers in Krabi Province, Thailand, using a questionnaire. The questionnaire consisted of four sections which included information on demographic characteristics, work-related characteristics, job stress, and MSDs. A total of 334 oil palm harvesting workers participated in the current study. The prevalence of MSDs during the past 12 months was 88.0%. Lower back MSDs had the highest (59.0%) 12-month prevalence among oil palm harvesting workers, followed by shoulder (37.1%) and neck (27.2%). Factors associated with lower back MSDs included type of task, heavy lifting, and job stress. Moreover, type of task, repetitive movement, and job stress were associated with shoulder and neck MSDs. The cutters had a higher risk of having shoulder and neck MSDs, primarily due to the fact that their work involved cutting the fresh fruit bunches from high up in the trees. The collectors had more back issues due to the heavy lifting. These findings showed the need to raise awareness, and to design guidelines and interventions to prevent MSDs in oil palm harvesting workers.

## 1. Introduction

Musculoskeletal disorders (MSDs) are common in various occupations and are one of the most challenging problems for public health worldwide [1,2,3,4,5]. MSDs may contribute to a decrease in quality of life [6,7], reduced work productivity [8], and cause chronic disability [9]. Agriculture is known as one of the most dangerous occupations, and these workers are at risk of developing MSDs due to the labor-intensive work. It has been reported that lifting and carrying heavy objects, prolonged or repeated stooping, and repetitive movement are the common risk factors in agriculture [10,11,12].

Oil palm farming is an important agricultural sector in Southeast Asia. Thailand is the world’s third largest oil palm producer, following Indonesia and Malaysia [13]. In Thailand, oil palm is an important economic crop due to its multiple utilities. Palm oil is used in various products, such as food items, commodities, and an alternative source of fuel energy. Moreover, the oil palm industry is a source of income and employment across Thailand, especially in the south and east [14]. In 2018, there were approximately 800,000 hectares of oil palm plantation area in Thailand. The total oil palm production in Thailand was more than 14.75 million tons, and Krabi is one of the top three provinces regarding oil palm plantation areas in the country [15].

Harvesting oil palm involves activities including cutting fresh fruit bunches (FFBs), collecting FFBs, and collecting loose fruits (LFs). The harvester usually uses a chisel to cut the FFBs from short palm trees, and a sickle to cut the FFBs from the tall palm trees. Cutting FFBs requires forceful exertions and involves awkward postures. The FFB collector uses a sharp metal skewer to collect the detached FFBs on the ground and carries them on their shoulder or loads the FFBs onto a wheelbarrow. The LF collector collects the LFs on the ground by sweeping them and loading them into a sack or a bucket. Collecting FFBs and LFs involves heavy lifting tasks, pushing and pulling, and awkward postures.

Although harvesting oil palm is labor intensive, the agricultural ergonomics related to harvesting oil palm have yet to be recognized. To date, there are only a few studies related to ergonomics in harvesting oil palm. Previous cross-sectional studies in Malaysia reported that the 12-month prevalence of MSDs among oil palm harvesting workers was extremely high (ranging from 86 to 99%) [8,16,17]. In addition, more recent studies in Thailand also reported high prevalence rates of MSDs in oil palm harvesting workers [18,19], with lower back pain having the highest prevalence among MSDs affecting oil palm harvesting workers [8,16,17,18,19].

At present, there has been limited research on the prevalence rate of MSDs and the associated risk factors in Thai oil palm harvesting workers. Such information will be useful to develop effective prevention and intervention strategies to promote health and safety in Thai oil palm harvesting workers. Therefore, the purpose of this study was to investigate the prevalence of MSDs and risk factors associated with MSDs in Thai oil palm workers during the harvesting stage.

## 2. Materials and Methods

### 2.1. Participants

This cross-sectional study was conducted in oil palm harvesting workers in Krabi Province, Thailand, from April to June 2018. The participants were selected from eight districts in Krabi Province. Oil palm harvesting workers in Krabi Province aged 18–60 years, having at least one year experience of harvesting oil palm, and working as oil palm harvesting workers at least six hours per day, five days per week, were included. Oil palm harvesting workers who had any history of major traumatic injuries, had been diagnosed with cancer or neurological disorders, or could not communicate in Thai were excluded. The sample size was calculated based on the prevalence rate of MSDs in oil palm harvesting workers from a previous study [8] with an estimated 20% non-response rate. The required sample size was 334. This study was approved by the Mahidol University Central Institutional Review Board (MU-CIRB COA. NO. 2018/005.1501).

### 2.2. Questionnaire

All participants were interviewed by trained interviewers using a questionnaire. The questionnaire consisted of four sections, which included information on demographic characteristics, work-related characteristics, job stress, and MSDs.

The first section was composed of questions regarding demographic characteristics (e.g., gender, age, body mass index (BMI), smoking behavior, drinking behavior, underlying disease, work experience, and income). The second section included questions about work-related characteristics, including work activities (4 items, e.g., types of task, working posture, and tools), work environment (4 items, e.g., perception about temperature, noise, and light), and ergonomic risk factors (8 items, e.g., using heavy tools, heavy lifting, repetitive movement, prolonged posture, and twisting of trunk). Questions regarding work activities were short answer and multiple choice questions. For the questions about perception of the work environment, there were options to choose between an inappropriate and an appropriate work environment. The questions regarding ergonomic risk factors were dichotomous questions (i.e., yes/no questions). The first and second sections of the questionnaire were developed based on literature reviews and information from in-depth interviews with ten experienced oil palm harvesting workers.

The third section included questions on job stress. This study used the Thai version of the 54-item Job Content Questionnaire (Thai-JCQ-54) [20], which was modified from Karasek’s Job Content Questionnaire [21]. The Thai-JCQ-54 consisted of 54 items measuring six dimensions: decision latitude (11 items), psychological job demand (12 items), physical job demand (6 items), job security (5 items), social support (8 items), and work hazards (12 items). All dimensions were scored ranging from 1 (strongly disagree) to 4 (strongly agree), except for the work hazards, which were scored ranging from 0 (no problem) to 3 (having many problems). The scores were dichotomized into two categories by median cutoff score: high score (≥median score) and low score (<median score). The job strain was determined by decision latitude and psychological job demand scores.

In the fourth section, the modified standardized Nordic questionnaire [22] was used. This section included questions regarding musculoskeletal discomfort in nine body parts (i.e., neck, shoulder, elbow, wrist/hand, upper back, lower back, hip/thigh, knee, and ankle/foot) during the past 12 months, and during the past week.

### 2.3. Statistical Analysis

Statistical data analysis was performed using Statistical Packages for the Social Sciences SPSS^®^ (version 21.0; IBM, Armonk, NY, USA). Descriptive statistics were used to obtain the mean, standard deviation, frequency, and percentage. Univariate logistic regression analysis was used to determine the association among demographic characteristics, work-related characteristics, job stress, and MSDs. Any variables with a *p*-value < 0.1 from the univariate logistic regression analysis, together with gender, age, and BMI, were included in the multivariate logistic regression analysis. A *p*-value < 0.05 was considered as significant for the final analysis.

## 3. Results

### 3.1. Demographic Characteristics

A total of 334 oil palm harvesting workers participated in this study. Table 1 summarizes the participants’ demographic characteristics. Participants consisted of 202 males (60.5%) and 132 females (39.5%) with a mean age of 39.4 ± 10.9 years. The mean BMI of the participants was 23.0 ± 4.2 kg/m^2^. The mean work experience and income per month of the participants were 9.3 ± 7.8 years and 10,847.3 ± 4589.5 Baht/month, respectively.

### 3.2. Work-Related Characteristics

Of the 334 participants, 118 participants (35.3%) were FFB cutters, 84 participants (25.2%) were FFB collectors, and 132 participants (39.5%) were LF collectors. The FFB cutters used a conventional chisel or sickle to cut the FFB. Of all the FFB cutters, 48.3% dropped their tool during cutting the FFB. For the FFB collectors, the majority of them used a sharp metal skew (90.5%) for collecting and transporting the FFBs. For the LF collectors, 69.7% worked in a stooped posture, and 30.3% worked in a squat posture. Table 2 presents the ergonomics risk factors related to work.

### 3.3. Job Stress

The results revealed that the majority of the participants perceived that they had high job control (55.1%), high psychological demand (51.2%), high physical demand (59.6%), high social support (96.7%), high job security (88.9%), and high work hazards (71.9%). Of all participants, 21.6% had a high-strain job, 25.4% had a low-strain job, 23.4% had a passive job, and 29.6% had an active job.

### 3.4. Prevalence of MSDs in All Oil Palm Harvesting Workers

The 12-month prevalence of MSDs was 88.0%. The region with the highest 12-month prevalence of MSDs in oil palm harvesting workers was lower back MSDs (59.0%), followed by shoulder MSDs (37.1%) and neck MSDs (27.2%). The 7-day prevalence of MSDs was 54.5%. Lower back MSDs showed the highest 7-day prevalence (26.6%), followed by shoulder MSDs (16.8%) and neck MSDs (13.8%) (Table 3).

### 3.5. Factors Associated with Lower Back MSDs in Oil Palm Harvesting Workers

In the univariate logistic regression analysis, type of task, heavy lifting, twisting of trunk, kneeling/squatting, and job stress were associated with lower back MSDs. The results from multivariable logistic regression analysis showed that the factors associated with lower back MSDs were type of task, heavy lifting, and job stress (Table 4). The FFB collectors and LF collectors had a 10.44 (adjusted OR = 10.44, 95% CI (5.00, 21.80)) and a 3.98 times (adjusted OR = 3.98, 95% CI (2.03, 7.81)) greater risk of developing lower back MSDs than the FFB cutters, respectively. The participants whose tasks involved heavy lifting were three times more likely to develop lower back MSDs than those whose tasks did not involve heavy lifting (adjusted OR = 3.00, 95% CI (1.38, 6.53)). Moreover, the participants who perceived their job as high strain had a 2.79 times greater odds of having lower back MSDs (adjusted OR = 2.79, 95% CI (1.41, 5.53)) than those who perceived their job as non-high strain.

### 3.6. Factors Associated with Shoulder MSDs in Oil Palm Harvesting Workers

The univariate logistic regression analysis revealed that type of task, heavy tool, repetitive movement, hand above shoulder, and job stress were factors associated with shoulder MSDs. In the multivariable logistic regression analysis, the following factors were associated with shoulder MSDs in oil palm harvesting workers: type of task, repetitive movement, and job stress (Table 5). The FFB cutters and FFB collectors had a 3.72 (adjusted OR = 3.72, 95% CI (1.77, 7.81)) and a 2.51 times (adjusted OR = 2.51, 95% CI (1.25, 5.06)) greater risk of developing shoulder MSDs than the LF collectors, respectively. The participants whose tasks involved repetitive movement were 1.96 times more likely to develop shoulder MSDs than those whose tasks did not involve repetitive movement (adjusted OR = 1.96, 95% CI (1.09, 3.52)). In addition, the chance of having shoulder MSDs was 3.39 times greater in the participants who perceived their job as high strain than those who perceived their job as non-high strain (adjusted OR = 3.39, 95% CI (1.86, 6.15)).

### 3.7. Factors Associated with Neck MSDs in Oil Palm Harvesting Workers

In the univariate logistic regression analysis, type of task, heavy tool, repetitive movement, hand above shoulder, and job stress were associated with neck MSDs. The results from multivariable logistic regression analysis showed that type of task, repetitive movement, and job stress were the factors associated with neck MSDs (Table 6). The FFB cutters had a 4.82 times greater chance of developing neck MSDs than the LF collectors (adjusted OR = 4.82, 95% CI (2.07, 11.24)). Moreover, the participants who performed repetitive movement had a 2.19 times higher risk of developing neck MSDs than those who did not (adjusted OR = 2.19; 95% CI (1.06, 4.51)). The participants who perceived their job to be high strain were 7.26 times more likely to have neck MSDs (adjusted OR = 7.26; 95% CI (3.55–14.86)).

## 4. Discussion

In this study, the 12-month prevalence rate of MSDs in any body region among oil palm harvesting workers was 88.0%. The extremely high 12-month prevalence rate of MSDs found in the current study was comparable with previous studies [8,16,18,19], but higher than another study by Henry and colleagues [23]. A systematic review by Osborne et al. also reported that the 1-year prevalence of MSDs among farmers was 76.9% [24]. Moreover, we also found that the 7-day prevalence of MSDs in the current study was 54.5%. The 7-day prevalence of MSDs in oil palm harvesting workers in this study was in line with previous studies in Malaysia [8,16]. However, the 7-day prevalence of MSDs reported in the current study was lower than in other studies in Thailand [18,19]. This might be due to the fact that this study was conducted during the off-peak season of FFB harvesting, while the other studies [18,19] were conducted during the peak season of FFB harvesting. The differences in the prevalence rate of MSDs across studies might be due to the differences in harvesting time of year, harvesting stage, and harvesting methods.

Furthermore, lower back MSDs were found to be the most prevalent (59.0%) MSDs during the past 12 months, followed by shoulder (37.1%) and neck (27.2%). These findings were in line with previous studies [18,23]. Lower back MSDs were reported to be the most prevalent 12-month prevalence of MSDs among oil palm harvesting workers in previous studies [8,16,17,18,19,23]. This study also found that the most common MSDs during the past week were lower back MSDs, which was in agreement with other studies [8,16,18]. The high prevalence rate of lower back MSDs in the oil palm harvesting workers might be due to their tasks involving the lifting and carrying of heavy loads, and repetitive movements in stooped postures.

In addition, we found that type of task and heavy lifting were associated with lower back MSDs among oil palm harvesting workers. The findings revealed that the FFB collectors and LF collectors had a greater chance of developing lower back MSDs than the FFB cutters. Workers who were involved with heavy lifting were more likely to report lower back MSDs. This could be explained by the job characteristics of the workers. The FFB collectors employed heavy lifting in awkward postures while carrying FFBs, and repetitive movement while in a stooped posture. A study by Deros et al. also asserted that the heavy and repetitive lifting of FFBs may contribute to lower back MSDs in FFB collectors [25]. For the LF collectors, their task included lifting and pulling the heavy sacks of LF, and prolonged or repetitive trunk bending.

Moreover, type of task and repetitive movement were found to be associated with shoulder and neck MSDs. The findings suggested that the FFB cutters had a greater chance of developing shoulder MSDs than the LF collectors. This may be due to the fact that the FFB cutters work with forceful exertion and repetitive movement of the shoulders. They also work with their hands above their shoulders while cutting FFBs from the tall oil palm trees. We also found that the FFB collectors were more likely to develop shoulder MSDs than the LF collectors. This may be attributed to the fact that the FFB collectors must carry the heavy FFBs on their shoulders repeatedly. Moreover, the FFB cutters were more likely to develop neck MSDs than the LF collectors. This could be explained by the working posture of the FFB cutters. In this study, the FFB cutters mostly harvested the FFBs from the tall oil palm trees. Therefore, they needed to continuously and repetitively tilt their head upward during the cutting of the FFB. The working posture observed in this study was consistent with previous studies [25,26].

Another factor that was found to be associated with lower back, shoulder, and neck MSDs was job stress. The findings showed that the majority of the participants perceived their job to have high job control, high psychological demand, high physical demand, high social support, high job security, and high work hazards. In addition, 29.6% of the participants perceived their job to be a high-strain job. Those who had high strain jobs were more likely to develop lower back, shoulder, and neck MSDs. This finding is consistent with previous studies [1,4,18,27,28,29,30]. Working under time pressure was found to be associated with lower back MSDs in oil palm harvesting workers [18]. Systematic reviews also reported the association between psychosocial factors and lower back pain [27,28]. Previous studies also showed that stress was associated with shoulder pain [4,30] and neck pain [29]. By contrast, other studies found no association between stress and MSDs [5,31].

Regarding the research implication for this study, the findings could provide information on the prevalence of MSDs and risk factors associated with MSDs among Thai oil palm harvesting workers, which is quite limited in Thailand. This information could be used to develop strategies and guidelines to promote safety and health among Thai oil palm harvesting workers. In addition, this study suggested that FFB cutters were more vulnerable to neck and shoulder MSDs due to the repetitive overhead work. Moreover, the collectors were prone to lower back MSDs from the heavy lifting from the ground. Therefore, ergonomically designed harvesting tools and machines are needed to reduce the awkward working posture and the risk of developing MSDs in oil palm harvesting workers. The findings of this study also raise awareness regarding the association between job stress and MSDs among oil palm harvesting workers.

However, there were several limitations in this study. First, this study was conducted in Krabi Province, Thailand; thus, the generalization of the findings might be limited. Second, this study was a cross-sectional study. Therefore, the causal relationship could not be established. Third, this study used a questionnaire, so it may be subject to recall bias. Fourth, there was no physical examination. Thus, the prevalence of MSDs was based on self-reports of the workers, which might be subjective.

## 5. Conclusions

This study suggests an extremely high prevalence of MSDs among Thai oil palm harvesting workers. The highest prevalence was of lower back MSDs, followed by shoulder MSDs and neck MSDs, respectively. In addition, types of task and job stress were associated with lower back MSDs, shoulder MSDs, and neck MSDs. There was also an association between heavy lifting and lower back MSDs. Moreover, repetitive movement was associated with shoulder and neck MSDs. The FFB cutters had a greater risk of developing shoulder and neck MSDs, primarily due to the overhead work involved in cutting the FFBs up in the trees. The collectors were more likely to suffer from lower back MSDs due to the heavy lifting. The findings from this study suggest that guidelines and training, such as exercise, proper working posture, and stress management, should be provided to the Thai oil palm harvesting workers. Moreover, ergonomically designed tools should be developed for promoting health and safety in oil palm harvesting workers.

## Figures and Tables

**Table 1:** Demographic characteristics of all participants (*n* = 334).

Variables	*n*	%
**Gender**		
Female	132	39.5
Male	202	60.5
**Age** (mean ± SD): 39.4 ± 10.9 years		
18–30	84	25.2
31–40	93	27.8
41–50	99	29.6
51–60	58	17.4
**Body Mass Index (BMI)** 23.0 ± 4.2 kg/m^2^		
<18.5 (Underweight)	27	8.1
18.5–22.9 (Normal)	161	48.2
23.0–24.9 (Overweight)	65	19.5
≥25.0 (Obese)	81	24.2
**Marital status**		
Single	32	9.6
Married	302	90.4
**Educational level**		
Primary school	224	67.1
Secondary school and higher	110	32.9
**Smoking**		
No	155	46.4
Yes	179	53.6
**Drinking**		
No	214	64.1
Yes	120	35.9
**Underlying disease**		
No	248	74.3
Yes	86	25.7
**Exercise**		
No	285	85.3
Yes	49	14.7
**Work status**		
Owner	23	6.9
Employee	311	93.1
**Experience** (mean ± SD): 9.3 ± 7.8 years		
≤10 years	226	67.7
>10 years	108	32.3
**Income** (mean ± SD): 10,847.3 ± 4589.5 Baht		
≤10,000 Baht	198	59.3
>10,000 Baht	136	40.7

**Table 2:** Ergonomics risk factors in oil palm harvesting workers (*n* = 334).

Variables	FFB Cutters (*n* = 118)		FFB Collectors (*n* = 84)		LF Collectors (*n* = 132)	
	*n*	%	*n*	%	*n*	%
**Heavy tool**						
No	13	11.0	21	25.0	78	59.1
Yes	105	89.0	63	75.0	54	40.9
**Heavy lifting**						
No	16	13.6	2	2.4	25	18.9
Yes	102	86.4	82	97.6	107	81.1
**Repetitive movement**						
No	21	17.8	19	22.6	65	49.2
Yes	97	82.2	65	77.4	67	50.8
**Prolonged posture**						
No	17	14.4	11	13.1	43	32.6
Yes	101	85.6	73	86.9	89	67.4
**Twisting of trunk**						
No	69	58.5	43	51.2	52	39.4
Yes	49	41.5	41	48.8	80	60.6
**Kneeling/Squatting**						
No	93	78.8	65	77.4	31	23.5
Yes	25	21.2	19	22.6	101	76.5
**Hand above shoulder**						
No	8	6.8	32	38.1	86	65.2
Yes	110	93.2	52	61.9	46	34.8
**Vibration**						
No	108	91.5	82	97.6	120	90.9
Yes	10	8.5	2	2.4	12	9.1

Abbreviations: FFB, fresh fruit bunch; LF, loose fruit.

**Table 3:** Prevalence of MSDs during the past 12 months and 7 days (*n* = 334).

Regions	During the Past 12 Months		During the Past 7 Days	
	*n*	%	*n*	%
Neck	91	27.2	46	13.8
Shoulder	124	37.1	56	16.8
Elbow	34	10.2	10	3.0
Wrist	43	12.9	17	5.1
Upper back	61	18.3	15	4.5
Lower back	197	59.0	89	26.6
Hip	30	9.0	11	3.3
Knee	45	13.5	24	7.2
Ankle	9	2.7	6	1.8
All body region	294	88.0	182	54.5

**Table 4:** Multivariate analysis of lower back MSDs during the past 12 months.

Variable	Adjusted OR	95% CI	*p*-Value
**Type of task**			
FFB cutters	1		
FFB collectors	10.44	5.00–21.80	<0.001 **
LF collectors	3.98	2.03–7.81	<0.001 **
**Heavy Lifting**			
No	1		
Yes	3.00	1.38–6.53	0.006 *
**Job stress**			
Non-high strain	1		
High strain	2.79	1.41–5.53	0.003 *

* Significant value *p* < 0.05; ** *p* < 0.001; OR: odds ratio; 95% CI: 95% confidence interval.

**Table 5:** Multivariate analysis of shoulder MSDs during the past 12 months.

Variable	Adjusted OR	95% CI	*p*-Value
**Type of task**			
FFB cutters	3.72	1.77–7.81	0.001 *
FFB collectors	2.51	1.25–5.06	0.010 *
LF collectors	1		
**Repetitive movement**			
No	1		
Yes	1.96	1.09–3.52	0.025 *
**Job stress**			
Non-high strain	1		
High strain	3.39	1.86–6.15	<0.001 **

* Significant value *p* < 0.05; ** *p* < 0.001; OR: odds ratio; 95% CI: 95% confidence interval.

**Table 6:** Multivariate analysis of neck MSDs during the past 12 months.

Variable	Adjusted OR	95% CI	*p*-Value
**Type of task**			
FFB cutters	4.82	2.07–11.24	<0.001 **
FFB collectors	0.48	0.18–1.24	0.129
LF collectors	1		
**Repetitive movement**			
No	1		
Yes	2.19	1.06–4.51	0.034 *
**Job stress**			
Non-high strain	1		
High strain	7.26	3.55–14.86	<0.001 **

* Significant value *p* < 0.05; ** *p* < 0.001; OR: odds ratio; 95% CI: 95% confidence interval.

## Data Availability

Not applicable.
